# An adaptable defense

**DOI:** 10.7554/eLife.56122

**Published:** 2020-03-30

**Authors:** Michael A Schelling, Dipali G Sashital

**Affiliations:** Roy J Carver Department of Biochemistry, Biophysics and Molecular Biology, Iowa State UniversityAmesUnited States

**Keywords:** CRISPR, bacteriophage, coevolution, bacterial immunity, bacteria, Other

## Abstract

The response of bacteria to the threat posed by phages depends on their local environment.

**Related research article** Pyenson NC, Marraffini LA. 2020. Co-evolution within structured bacterial communities results in multiple expansion of CRISPR loci and enhanced immunity. *eLife* **9**:e53078. doi: 10.7554/eLife.53078

Many bacteria use a system known as CRISPR-Cas to defend themselves against infection by viruses called phages. This system protects the bacterial cell by taking a short length of DNA from the phage and inserting this 'spacer' into its own genome. If the bacterial cell becomes re-infected, the spacer allows the cell to recognize the phage and stop it from replicating by cutting and destroying its DNA. Bacteria with these spacers survive infections and pass their spacers on to their progeny, creating a population that is resistant to the phage.

Phage populations, however, can also adapt and evade bacterial CRISPR-Cas systems. For example, if a phage develops a random mutation in the region targeted by the spacer, it may become undetectable by CRISPR-Cas, leaving it free to replicate and infect other cells (Barrangou et al., 2007; Deveau et al., 2008). Bacteria can combat these phages by creating multiple spacers that target different regions of the phage genome (van Houte et al., 2016; Figure 1). However, previous studies have shown that bacteria rarely acquire multiple spacers (Heler et al., 2015). Now, in eLife, Nora Pyenson and Luciano Marraffini from the Rockefeller University report that the number of spacers each bacterial cell acquires depends on its local environment (Pyenson and Marraffini, 2020).

**Figure 1.**
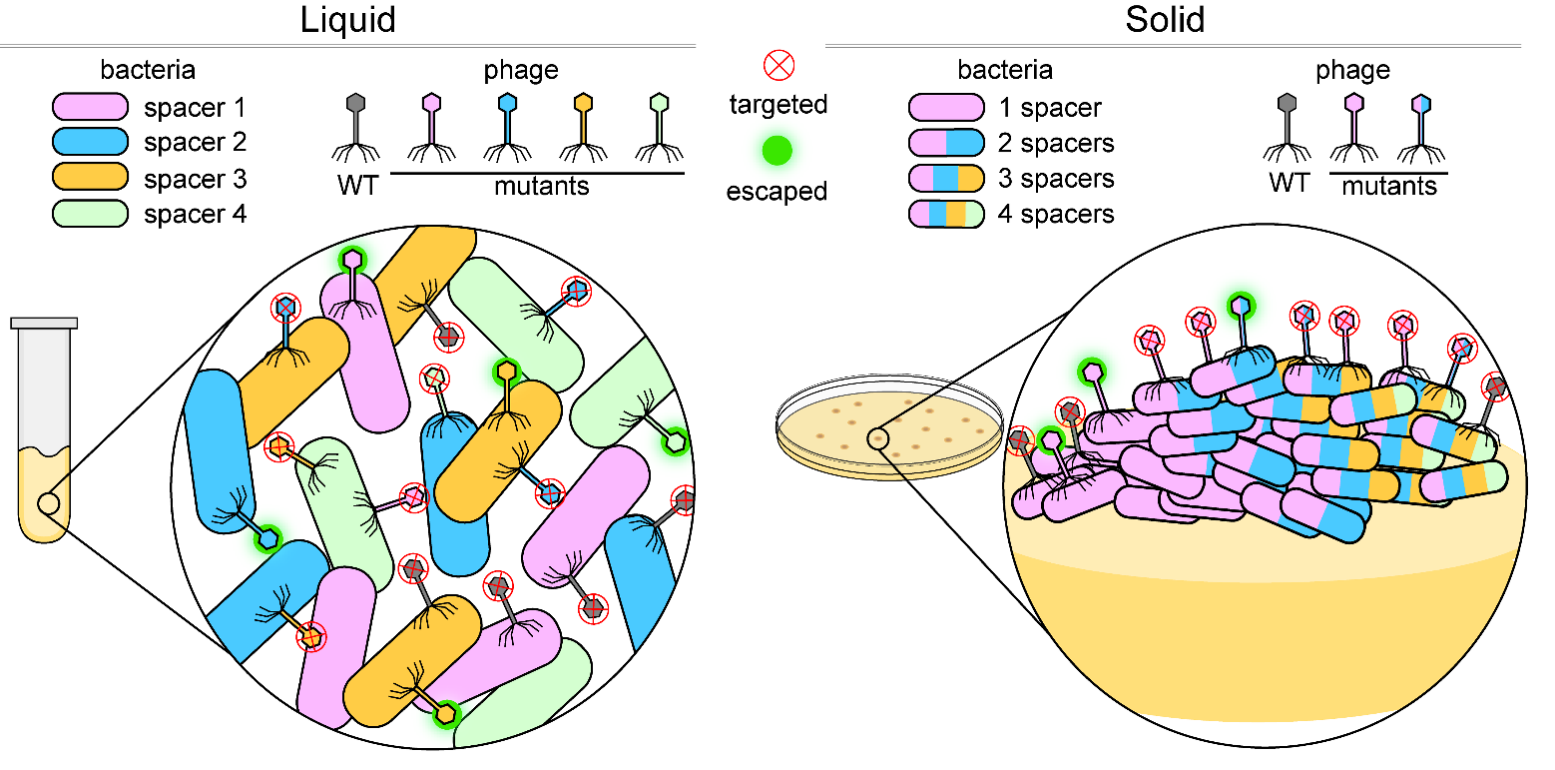
Bacteria versus viruses called phages. To defend themselves against phages, bacteria (colored capsules) acquire a region of a phage genome and insert it into their own genome as a 'spacer'. When growing in a liquid environment (left), individual bacterial cells usually acquire a single spacer that targets just one region of the wild-type (WT) phage (shown in grey). In the figure, each bacterial cell has one of four different spacers (shown in blue, green, orange and pink). However, phages mutate in an effort to bypass these defenses: a mutation in the region of the phage genome corresponding to, say, a blue spacer means that the phage can attack and escape the defense of bacteria with blue spacers (fuzzy green circle), but not bacteria with green, orange or pink spacers (red X inside a circle). When growing on a solid surface (right), if an individual cell acquires, say, a pink spacer, it will go on to form a colony of phage resistant cells (inset). If a phage gains a mutation in the region targeted by the pink spacer, the phage will escape detection. In order to stay protected, some bacterial cells within the colony acquire multiple spacers (multi-colored bacterial cells) and can fight off various mutant phages.

Image credit: Dipali Sashital (CC BY 4.0)

Bacteria grown in liquid culture rarely have multiple spacers. Pyenson and Marraffini hypothesized that this is because bacterial cells move more freely when in this environment and are thus able to work together to defend themselves (Figure 1, left). This limits the need for individual cells to have multiple spacers in order to be protected. To test this theory, Pyenson and Marraffini investigated what happens to infected bacteria that are grown on a solid medium where cell movement is restricted. They found that most cells died, but those that acquired resistance formed separate colonies. Further experiments showed that compared to bacteria grown in liquid culture, bacterial cells in the resistant colonies had often acquired multiple spacers and were able to fight off phages with other mutations (Figure 1, right).

However, it still remained unclear what drives bacteria to acquire multiple spacers. It was previously shown that acquiring an initial spacer can drive the addition of subsequent spacers through a process called priming (Datsenko et al., 2012; Nussenzweig et al., 2019). Pyenson and Marraffini found that a disproportionate number of second spacers were taken from DNA regions located close to the initial spacer, which is a hallmark of priming. This suggests that priming enables immobile bacteria to survive mutated phages that have escaped detection by allowing the bacteria to create multiple spacers.

Cells in these resistant colonies arranged themselves into unusually shaped sectors, with the number and type of spacers present varying between cells in each sector. It seems that when bacteria are immobilized, individual cells within the colony must acquire more spacers to resist infection by the mutated phage (Figure 1, right). The new multi-spacer cells then outgrow the rest of the colony, forming these unusually shaped sectors. These findings suggest that bacteria cooperate and share the spacer load in a liquid environment. On a solid surface, on the other hand, the bacteria are more independent, and if a cell becomes immune to a mutated phage, it will form a new colony sector with its progeny.

In nature, bacteria are often attached to surfaces. For example, some bacteria live at the bottom of bodies of water, and these bacterial communities may be regularly targeted by phages (Tuson and Weibel, 2013). Bacteria also gather on biological surfaces when preparing to invade other organisms. For example, the bacteria *Streptococcus* preferentially bind and form colonies on mucus membrane surfaces in the human body before infecting epithelial cells (Beachey, 1981). The study by Pyenson and Marraffini reveals how different types of environments may influence the way that bacteria and phages respond to each other and synchronously evolve over time.

CRISPR-Cas systems are exceptionally diverse and use a variety of mechanisms to defend bacteria against infection. Further experiments studying these systems in a variety of environmental contexts will be important to help explain the diverse mechanisms of CRISPR-Cas systems.

## References

[bib1] Barrangou R, Fremaux C, Deveau H, Richards M, Boyaval P, Moineau S, Romero DA, Horvath P (2007). CRISPR provides acquired resistance against viruses in prokaryotes. Science.

[bib2] Beachey EH (1981). Bacterial adherence: adhesin-receptor interactions mediating the attachment of bacteria to mucosal surface. Journal of Infectious Diseases.

[bib3] Datsenko KA, Pougach K, Tikhonov A, Wanner BL, Severinov K, Semenova E (2012). Molecular memory of prior infections activates the CRISPR/Cas adaptive bacterial immunity system. Nature Communications.

[bib4] Deveau H, Barrangou R, Garneau JE, Labonté J, Fremaux C, Boyaval P, Romero DA, Horvath P, Moineau S (2008). Phage response to CRISPR-encoded resistance in *Streptococcus thermophilus*. Journal of Bacteriology.

[bib5] Heler R, Samai P, Modell JW, Weiner C, Goldberg GW, Bikard D, Marraffini LA (2015). Cas9 specifies functional viral targets during CRISPR-Cas adaptation. Nature.

[bib6] Nussenzweig PM, McGinn J, Marraffini LA (2019). Cas9 cleavage of viral genomes primes the acquisition of new immunological memories. Cell Host & Microbe.

[bib7] Pyenson NC, Marraffini LA (2020). Co-evolution within structured bacterial communities results in multiple expansion of CRISPR loci and enhanced immunity. eLife.

[bib8] Tuson HH, Weibel DB (2013). Bacteria-surface interactions. Soft Matter.

[bib9] van Houte S, Ekroth AK, Broniewski JM, Chabas H, Ashby B, Bondy-Denomy J, Gandon S, Boots M, Paterson S, Buckling A, Westra ER (2016). The diversity-generating benefits of a prokaryotic adaptive immune system. Nature.

